# Diatomaceous Earth Supplementation as a Bioavailable Silicon Source Modulates Postprandial Lipid Metabolism in Healthy Female Rats

**DOI:** 10.3390/nu17152452

**Published:** 2025-07-28

**Authors:** Rocío Redondo-Castillejo, Marina Hernández-Martín, Jousef Ángel Issa-García, Aránzazu Bocanegra, Alba Garcimartín, Adrián Macho-González, Sara Bastida, Francisco J. Sánchez-Muniz, Juana Benedí, M. Elvira López-Oliva

**Affiliations:** 1 Pharmacology, Pharmacognosy and Botany Department, Pharmacy School, Complutense University of Madrid, 28040 Madrid, Spain; roredond@ucm.es (R.R.-C.); a.garcimartin@ucm.es (A.G.); jbenedi@ucm.es (J.B.); 2 Departmental Section of Physiology, Pharmacy School, Complutense University of Madrid, 28040 Madrid, Spain; marinh04@ucm.es (M.H.-M.); jissa@ucm.es (J.Á.I.-G.); 3 Nutrition and Food Science Department, Pharmacy School, Complutense University of Madrid, 28040 Madrid, Spain; amacho@ucm.es (A.M.-G.); sbastida@ucm.es (S.B.); frasan@ucm.es (F.J.S.-M.)

**Keywords:** diatomaceous earth supplementation, silicon, postprandial triglyceridemia, intestinal and hepatic lipid metabolism, female rats, intestinal mucosal barrier

## Abstract

Background/Objectives: Diatomaceous earth (DE), a natural substance rich in amorphous silica and recognized as a food additive, is gaining attention as a dietary silicon supplement. However, its bioavailability and impact on lipid digestion and absorption remain poorly characterized. This study aimed to investigate silicon bioavailability after short-term DE supplementation and its effects on postprandial glycemia and triglyceridemia, the expression of lipid metabolism-related proteins, and the modulation of the intestinal mucosal barrier. Methods: Female Wistar rats received daily oral supplementation of DE (equivalent to 2 or 4 mg silicon/kg body weight) for one week. Silicon digestibility, excretion, and hepatic accumulation were quantified. Postprandial glycemia and triglyceridemia were monitored. Lipid profile was analyzed by HPSEC in gastric and intestinal contents. Jejunal morphology and mucin-secreting cells were assessed histologically. Lipid metabolism markers were evaluated by immunohistochemistry and Western blot in both intestinal and hepatic tissues. Results: DE supplementation enhanced silicon absorption and increased hepatic levels. Fecal output and moisture content were also elevated, especially at the higher dose. DE significantly reduced postprandial triglyceridemia and consequently increased luminal triglyceride retention. These changes were associated with decreased jejunal levels of IFABP, ACAT2, and MTP, as well as reduced hepatic levels of MTP and LDLr, alongside increased levels of ABCG5/G8 and LXRα/β, indicating a partial blockage of lipid absorption and enhanced cholesterol efflux. The effects on the intestinal barrier were evidenced by villi shortening and an increase in mucin-producing cells. Conclusion: Food-grade DE is a bioavailable source of silicon with hypolipidemic potential, mainly by reducing intestinal lipid absorption. This is supported by lower postprandial triglycerides, increased luminal lipid retention, and decreased expression of lipid transport proteins. The study in healthy female rats underscores the importance of sex-specific responses and supports DE as a dietary strategy to improve lipid metabolism.

## 1. Introduction

Diatomaceous earth (DE), primarily composed of amorphous silica, has long been utilized in agriculture and industry. In the food sector, it is authorized as a food additive (E551) due to its anti-caking and moisture-retention properties [1,2] and is approved for human consumption by regulatory authorities [3,4]. While its technological applications are well established, its nutritional potential, particularly as a source of dietary silicon, has recently attracted scientific interest.

Silicon is an essential trace element involved in bone formation, connective tissue integrity, and cardiovascular health [5]. Its bioavailability depends largely on its chemical form, with orthosilicic acid (OSA) being very absorbable [6,7]. Although amorphous silica is water-insoluble, it can become hydrated in the gastrointestinal tract and convert into OSA, allowing systemic absorption [8,9]. Studies have shown that silicon, once absorbed, is cleared from plasma, and primarily excreted via the kidneys, with transient accumulation in soft tissues such as the liver [5,10]. Beyond its nutritional relevance, growing interest has emerged around the role of dietary silicon in metabolic health.

Postprandial hypertriglyceridemia, along with postprandial hyperglycemia, is recognized as a marker of altered lipid and glucose metabolism and is a key risk factor for cardiovascular and metabolic diseases [11]. Nutritional strategies aimed at reducing postprandial lipemia are gaining attention. Alterations in lipid absorption can influence gastric emptying and intestinal transit, thereby affecting both local and systemic metabolic homeostasis [12]. Given the increasing interest in functional dietary ingredients to modulate lipid metabolism, DE stands out as a potential hypolipidemic agent. Preliminary evidence suggests that distinctive physicochemical properties of silicon may reduce fat digestibility and improve postprandial lipemia. Garcimartín et al. [13] reported that organic silicon supplementation lowered both postprandial triglycerides and glucose levels in healthy rats. These effects were associated with modulation of intestinal glucose and lipid digestive enzymes and transporters. Similarly, Peluso and Schneeman [14] reported that dietary supplementation with food-grade silicon dioxide exerted hypocholesterolemic effects in cholesterol-fed rats. More recently, the consumption of silicon-enriched restructured meat (Si-RM) significantly improved lipid profiles in a late-stage type 2 diabetes mellitus (T2DM) rat model, confirming its anti-hypercholesterolemic effect [15]. Notably, most of these studies have been conducted exclusively in male animals, leaving a critical gap in understanding silicon’s effects in females. Female rodents typically exhibit a blunted postprandial TG response compared to males, which can limit the observable efficacy of lipid-lowering compounds [16]. Therefore, demonstrating a hypolipidemic effect in females not only underscores the physiological relevance of the intervention but also enhances its translational relevance.

In parallel, other mechanisms that could interfere with lipid absorption include disrupting micelle formation, hindering enzyme–substrate interactions, or altering intestinal transport pathways [17]. Recent studies suggested that silicon may impact lipid absorption not only through its structural physical interaction with lipids but also by modifying intestinal morphology and enhancing mucin production [18,19]. Increased mucin secretion may act as a diffusion barrier, limiting micelle–epithelial contact and thus reducing lipid uptake [20]. Moreover, the consumption of Si-RM has been shown to restore intestinal barrier integrity and attenuate oxidative stress and inflammation, further supporting its role in improving metabolic and intestinal health [21]. At the molecular level, the beneficial effects of Si-RM consumption on intestinal structure appear linked to the regulation of cholesterol homeostasis through modulation of intestinal lipid transporters, hepatic lipid metabolism, and enterohepatic bile acid circulation [16,18,19,22].

Whether the supplementation of DE ensures silicon bioavailability and reproduces the metabolic effects mentioned remains unexplored. Moreover, emerging research suggests that DE, composed of a high percentage of silicon dioxide, may exert functional roles beyond its silicon content, particularly influencing lipid metabolism and gastrointestinal physiology [5]. Due to its high porosity and absorptive capacity, DE could alter lipid digestion by modulating intestinal lumen properties and potentially interfering with micelle formation. Cofrades et al. [23] demonstrated that DE added to gelled emulsions reduced lipid digestibility during in vitro digestion, likely by stabilizing the emulsion structure and limiting lipase access. However, the underlying mechanisms remain poorly known. In this context, and considering that the biological effects of DE are not solely attributable to its silicon content but also to its structural properties, we investigated the physiological impact of short-term oral food-grade DE supplementation in healthy female rats, focusing on silicon bioavailability, postprandial responses, and molecular regulation of lipid absorption and metabolism at both the intestinal and hepatic levels.

## 2. Materials and Methods

### 2.1. Animals and Treatments

Eighteen healthy two-month-old female Wistar rats (Harlan S.L., Barcelona, Spain), weighing 150–200 g, were housed at the Animal Experimentation Center of Alcalá University, Madrid, Spain (registration number ES280050001165), grouped in pairs under controlled environmental conditions (22.3 ± 1.9 °C, 12 h light/dark cycle). The experimental protocol was approved by the Spanish Science and Technology Advisory Committee (project PID2019-103872RB-I00 and AEI/10.13039/501 100 011 033) and by the Ethics Committee at Complutense University of Madrid (UCM) (protocol code: PROEX 315.2/21, approval date: 18 October 2021). All procedures adhered to the Directive 2010/63/EU on the protection of animals used for scientific purposes and complied with the ARRIVE guidelines, ensuring ethical standards and transparency in animal research.

The food-grade DE preparation used in this study was Silicon Diatomeas^®^—Diatomaceous Earth, provided by Vitality Gesf S.L. (Valencia, Spain). According to the supplier’s specifications, the product contained 92% SiO_2_, corresponding to a 43% silicon content measured by inductively coupled plasma optical emission spectrometry (ICP-OES), performed at the CAI of Earth Sciences and Archaeometry, Faculty of Geological Sciences, UCM. Based on the silicon content of the DE preparation, the administered doses were adjusted to ensure the daily supply of 2 mg (DE2) or 4 mg (DE4) of silicon/kg body weight. These doses were selected according to previous studies reporting metabolic effects at comparable levels of silicon supplementation [13,15,18,19,21,22,24,25]. DE suspensions were freshly prepared in distilled water immediately before administration.

Rats were provided ad libitum access to water and a standard rodent diet (AIN-93M, Sodispan, Madrid, Spain). After a 5-day adaptation period, rats were randomly divided into three groups (*n* = 6) and administered daily by oral gavage for 7 days as follows: control group (C, administered with tap water), DE2 group (administered a dose containing 2 mg silicon/kg body weight/day), or DE4 group (administered a dose containing 4 mg silicon/kg body weight/day). Food intake and body weight were measured at the beginning and the end of the experimental period. On the fifth day of the treatments, oral tolerance tests for glucose (OGTT) and triglycerides (TG)(OTTT) were conducted. Following a 12 h overnight fast, rats were orally gavaged with 2 g/kg body weight of glucose and 1 mL of olive oil, administered either alone (C group) or in combination with the designated DE treatment (DE2 group or DE4 group). Blood samples were drawn from the tail vein at fasting (0 min) and at 30, 60, 120, 180, and 240 min post-administration. Blood glucose levels were measured using a glucometer (Accu-Chek Aviva; Roche Diagnostics, Mannheim, Germany), while TG concentrations were tested spectrophotometrically (SPECTROstar Omega, Ortenberg, Germany) by an enzymatic colorimetric assay (GPO-POD, Spinreact S.A., Barcelona, Spain). All measurements were performed duplicate for accuracy and reproducibility. The area under the incremental curve (AUIC) was calculated using the trapezoidal rule to assess the postprandial response over 0–4 h (AUIC_0–4_). This response was further segmented into early (AUIC_0–2.5_) and late (AUIC_2.5–4_) phases. To characterize the dynamics of lipid absorption and clearance during the postprandial period, TG absorption (AR) and removal rates (RR) were calculated as the slopes of the curves from 0 to peak time and from peak time to 4 h, respectively, to characterize the dynamics of lipid absorption and clearance during the postprandial period [16]. The TG peaks were classified in early peaks (high value until 2.5 h) and late peaks (high value after 2.5 h). Twenty-four hours before euthanasia, rats were placed individually in metabolic cages to collect urine and feces. Samples were immediately stored at −20 °C until further analysis. On the final day of the experiment, rats were administered 1 mL of olive oil by oral gavage, with or without the assigned dose of DE, and euthanized 2.5 h later under 5% isoflurane anesthesia, by exsanguination via the descending aorta using a heparinized syringe. Blood was centrifugated at 1500× *g* for 15 min at 4 °C to obtain plasma. After the euthanasia, gastric and intestinal contents were obtained by perfusing with 0.9% saline solution and stored at −80 °C until analysis. After that, the stomach, small intestine, and liver were rinsed in 0.9% saline solution, weighed and immediately dissected and frozen for subsequent analyses.

### 2.2. Determination of Silicon Content in Food, Water, Feces, Urine, Plasma, and Liver

Silicon content from the food, water, liver, feces, urine, plasma, and liver were analyzed. Firstly, an acid digestion with nitric acid (HNO_3_), hydrogen peroxide (H_2_O_2_), and hydrofluoric acid (HF) was performed in a high-pressure microwave digestion system to ensure complete dissolution of the sample matrix. Silicon concentrations were quantified by inductively coupled plasma optical emission spectrometry (ICP-OES) (CAI of Earth Sciences and Archaeometry, Faculty of Geological Sciences, UCM, Madrid, Spain) or atomic absorption spectroscopy (FAAS) (the Institute of Food Science, Technology and Nutrition (ICTAN-CSIC), Madrid, Spain), with calibration performed using standard solutions and validation with an international certified reference material. Triplicate analyses ensured accuracy and reproducibility. Only well-preserved, contamination-free samples were included in the final quantification. All procedures were conducted under controlled conditions, enabling robust detection and comprehensive assessment of silicon in diverse biological matrices.

### 2.3. Assessment of Silicon Digestibility and Absorption Coefficients

The silicon apparent digestibility coefficient (SiADC) was calculated using the formula:

SiADC (%) = (Silicon intake − Silicon in feces)/Silicon intake) × 100,(1)

where silicon intake was estimated based on the silicon concentration in the diet and drinking water, and fecal silicon was determined by multiplying fecal output by its silicon concentration [26].

Silicon absolute absorption coefficient (SiAAC) was determined by measuring dietary silicon intake, considering its content in the diet and water, and fecal and urinary silicon excretion. It was calculated using the following formula [26]:

SiAAC (mg) = Silicon intake (mg) − Silicon fecal excretion (mg) − Silicon urinary excretion (mg).(2)

### 2.4. Collection and Characterization of Gastric and Intestinal Lipid Content

Lipids from gastric and intestinal contents were extracted with a chloroform/methanol mixture (1:1, *v*/*v*) following a modification of the Bligh and Dyer’s method [27]. High-performance size-exclusion chromatography (HPSEC) was performed to determine the lipid composition following the methodology of Dobarganes et al. [28]. The analysis was conducted using a high-performance liquid chromatograph (1100 series, Agilent Technologies, Madrid, Spain) equipped with a 20 μL sample loop, a refractive index detector (1260 Infinity, Agilent Technologies), and two serially connected 300 mm × 7.5 mm (inside diameter) PL gel columns (particle sizes of 0.01 μm and 0.05 μm, Agilent, Bellefonte, PA, USA). The mobile phase was high-performance liquid chromatography-grade tetrahydrofuran (THF) at a flow rate of 1 mL/min, and the column was operated at 30 °C. For each analysis, 15 mg of extracted fat diluted in 1 mL of THF was injected. The system was calibrated using lipid standards, including TG, diglycerides (DG), monoglycerides (MG), and free fatty acids (FFA), to ensure accurate quantification of lipid fractions. The total fat content and lipid profiles were quantified as g/100 g of fat. To ensure precision and reproducibility, all samples were analyzed in triplicate, and the system was calibrated daily.

### 2.5. Morphometric Measurements

Samples from jejunum were fixed in 10% formaldehyde, embedded in paraffin, and sectioned at a thickness of 5 µm. Villi height and crypt depth were measured on hematoxylin and eosin (H&E)-stained sections, and at least 20 well-aligned villi and crypts per rat were analyzed. Villus height was determined by measuring the distance from the villus-crypt junction to the villus tip, and crypt depth was determined by the distance from villus-crypt junction to basal crypt cells. The results were expressed as the villus height to crypt depth ratio, which provides an index of intestinal structural integrity and absorptive capacity [29]. Images were captured using a Leica DM LB2 light microscope equipped with a Leica DFC 320 camera (Leica, Madrid, Spain) and analyzed with ImageJ software (Fiji ImageJ, version 1.54j, NIH, USA).

### 2.6. Detection and Quantification of Intestinal Mucins

To evaluate mucin production in the jejunal epithelium, histochemical staining was performed using periodic acid-Schiff (PAS) for neutral mucins and alcian blue (AB, pH 2.5) for acidic mucins. Paraffin-embedded jejunal sections (5 μm thick) were deparaffinized, rehydrated, and stained according to standard protocols. For each sample, ten well-oriented villus or crypt units were selected, and the number of PAS-positive (PAS+) and AB-positive (AB+) goblet cells was counted under a light microscope (Leica, Madrid, Spain) at 400× magnification. The PAS/AB+ cells index was used to assess the relative abundance of neutral versus acidic mucins [30].

### 2.7. Immunohistochemical Staining

Immunohistochemical analysis was performed on jejunum and liver sections fixed in 10% paraformaldehyde in 0.1 M phosphate buffer (pH 7.4) and embedded in paraffin. Sections (5 µm thick) were deparaffinized, rehydrated, and treated with 3% hydrogen peroxide to block endogenous peroxidase activity. After antigen retrieval with citrate buffer (pH 6.0) and blocking, sections were incubated overnight at 4 °C with the following primary antibodies: anti-NPC1L1 (sc-166802), anti-IFABP (sc-374482), anti-ACAT2 (sc-293307), anti-MTP (sc-515742), anti-LDLr (sc-18823), and anti-LXR (sc-377260) (all from Santa Cruz Biotechnology, Quimigen, Madrid, Spain); and anti-ABCG5 (orb5656), anti-ABCG8 (orb228808), and anti-ABCA1 (orb526545) (all from Biorbyt, Cambridge, UK). After incubation with biotinylated secondary antibodies and horseradish peroxidase-conjugated streptavidin-biotin staining, the reaction was visualized with 3,3′-diaminobenzidine (DAB) and counterstained with hematoxylin (Sigma-Aldrich, Madrid, Spain). Protein levels were quantified as an immunoreactivity score (IRS) based on staining intensity: weak (1), moderate (2), diffuse (3), or intense (4). Images from 10 fields per section (200× magnification) were analyzed using ImageJ software (Fiji ImageJ, version 1.54j, NIH, New York, NW, USA). Histological slides were independently evaluated in a blinded manner by two researchers, each without access to the other’s assessments or to data from other experimental outcomes at the time of analysis

### 2.8. Western Blot Analysis

Total proteins were extracted from jejunum or liver tissues using lysis buffer and quantified by the DS protein assay (BioRad, Madrid, Spain). Approximately 30–60 µg of total protein was separated by SDS-PAGE and transferred onto PVDF membranes. (Millipore, Billerica, MA, USA). Membranes were blocked with 5% skimmed milk for 1 h at room temperature, followed by overnight incubation at 4 °C with primary antibodies: anti-ACAT2 (sc-293307) and anti-MTP (sc-515742) (both from Santa Cruz Biotechnology, Quimigen, Madrid, Spain), and anti-ABCG8 (orb228808) (from Biorbyt, Cambridge, UK). After incubation with horseradish peroxidase-conjugated secondary antibodies, membranes were visualized using the ECL Select-kit (GE Healthcare, Madrid, Spain) and ImageQuant LAS 500 system (GE Healthcare, Madrid, Spain). Ponceau S staining (Sigma-Aldrich, Madrid, Spain) was used as a loading control. Protein levels were quantified using ImageQuant software (Version 5.0, GE Healthcare Life Sciences, Madrid, Spain).

### 2.9. Statistical Analysis

Results presented in tables and figures were expressed as mean ± standard deviation (SD) or standard error of the mean (SEM), respectively. Homogeneity of variances was assessed using Levene’s test. For multiple group comparisons, one-way analysis of the variance (ANOVA) was followed by the Bonferroni test when variances were homogeneous, or the Tamhane test when variances were not homogeneous. Non-parametric variables were analyzed using the Kruskal–Wallis test, followed by the Benjamini–Krieger–Yekutieli post hoc correction for multiple comparisons. Correlations between scores and parameters were determined using Pearson’s or Spearman’s correlation coefficients. Statistical significance was set at *p* < 0.05. Data analysis was performed using SPSS (version 28.0, SPSS Inc., Chicago, IL, USA), and graphs were generated using GraphPad Prism (version 8, GraphPad Software Inc., La Jolla, CA, USA).

## 3. Results

### 3.1. Ponderal Parameters, Hepatic, Fecal and Urine Silicon Contents, and Apparent Digestibility Silicon Absolute Absorption Coefficients

The results for ponderal parameters, as well as hepatic, fecal, and urine silicon contents, silicon digestibility coefficients, and absolute absorption are summarized in Table 1. No significant differences were found in body weight gain, stomach, small intestine and liver weights, and small intestine length across groups (*p* > 0.05). No significant differences were observed between groups in daily food intake or water consumption. Additionally, 24 h urine volume remained unaffected by DE administration (*p* > 0.05). However, fecal dry matter was significantly higher in the DE4 group compared to the C group (+29.5%, *p* = 0.045), while no significant difference was found between the C and DE2 groups (*p* > 0.05). The percentage of fecal moisture was also significantly higher in the DE4 group compared to both the control (+10.3%, *p* = 0.021) and DE2 (+8.9%, *p* = 0.050) groups. These results indicate a dose-dependent effect of DE on fecal output and moisture.

In contrast, hepatic silicon content significantly increased in the DE2 (+467.7%, *p* < 0.001) and DE4 (+332.8%, *p* < 0.01) groups compared to the C group (*p* < 0.001). Although the DE2 group exhibited the highest hepatic silicon concentration (10.73 ± 3.40 µg/g), no significant differences were observed compared to the DE4 group. Notably, silicon was not detectable in plasma samples collected at the time of sacrifice in any of the groups. Fecal silicon content was significantly higher in the DE2 (+101.8%, *p* = 0.01) and DE4 (+112.6%, *p* < 0.01) groups relative to the C group (*p* < 0.001). The SiADC (DE2 vs. C: +442.5%, *p* = 0.02; DE4 vs. C: +712.9%, *p* = 0.0002) and SiAAC (DE2 vs. C: +519.4%, *p* = 0.002; DE4 vs. C: +1080.6%, *p* < 0.00001) showed a clear dose-dependent increase.

### 3.2. Postprandial Glycemia and Triglyceridemia

**Figure 1:**
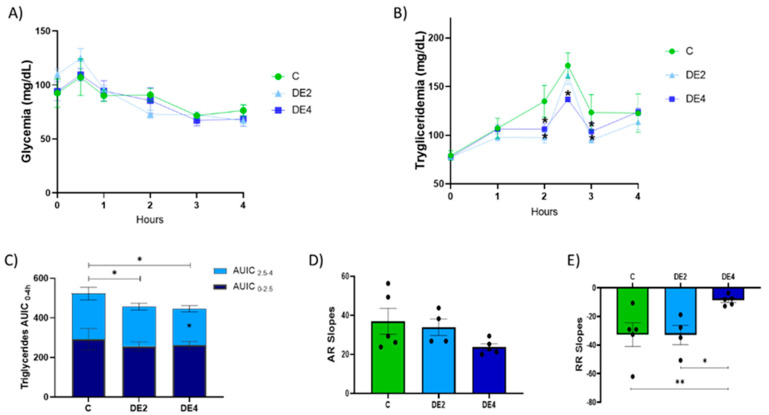
Tolerance tests for glucose (OGTT) and triglycerides (OTTT) after oral administration of glucose (2 g/kg body weight) and olive oil (1 mL) in healthy female Wistar rats. Data are expressed as mean ± SEM. C: rats administered daily with tap water; DE2: rats supplemented with 2 mg silicon/kg body weight/day; DE4: rats supplemented with 4 mg silicon/kg body weight/day. Blood glucose and triglyceride levels (mg/dL) were plotted against time (hours). (**A**) Time-course of plasma glucose concentrations (mg/dL) measured from 0 to 4 h after the glucose challenge. (**B**) Time-course of plasma triglyceride concentrations (mg/dL) measured from 0 to 4 h after the lipid challenge. (**C**) Area under the incremental curve (AUIC0–4) for postprandial TG response. (**D**) Triglyceride absorption rate (AR). (**E**) Triglyceride removal rate (RR). * indicates statistically significant differences between groups (* *p* < 0.05, ** *p* < 0.01) determined by one-way ANOVA followed by Bonferroni or Tamhane post hoc test, as appropriate.

Figure 1 displays the time course of postprandial plasma glucose (Figure 1A) and triglyceride (TG) concentrations (Figure 1B), along with the area under the incremental curve (AUIC_0–4_, AUIC_0–2_._5_, AUIC_2_._5–4_) (Figure 1C), the triglyceride absorption rate (AR) (Figure 1D), and the removal rate (RR) (Figure 1E) in rats subjected to oral tolerance tests (OGTT and OTTT) following DE supplementation at two doses (2 or 4 mg silicon/kg body weight/day) or tap water.

No significant differences were observed in postprandial glucose levels among groups (*p* < 0.05). In contrast, DE supplementation induced a dose-dependent reduction in postprandial TG concentrations.

Specifically, the DE2 group showed significant reductions in plasma TG levels at 2 h (−27.8%, *p* = 0.010) and 3 h (−22.3%, *p* = 0.028), while the DE4 group exhibited sustained reductions at 2 h (−21.2%, *p* = 0.039), 2.5 h (−13.0%, *p* = 0.049), and 3 h (−15.9%, *p* = 0.021) compared with the control group.

As shown in Figure 1C, both DE2 and DE4 significantly decreased AUIC_0–4_ relative to controls, indicating an overall attenuation of the postprandial TG response. Additionally, the DE4 group showed a specific reduction in AUIC_2_._5–4_ (−19.5%, *p* = 0.033), highlighting a dose-dependent effect of DE on the postprandial lipemic response.

While no significant differences were found in the ascending slope (AR; Figure 1D), the DE4 group exhibited a significantly flatter descending slope (RR; Figure 1E) compared to both the control (−39.6%, *p* = 0.006) and DE2 groups (−35.9%, *p* = 0.018), suggesting that DE supplementation modifies the dynamics of postprandial TG clearance.

### 3.3. Gastric and Intestinal Contents and Lipid Composition

**Figure 2:**
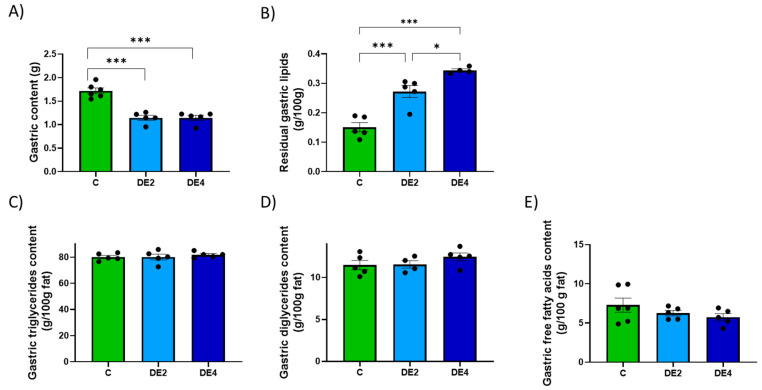
Gastric content and lipid composition after 2.5 h of an oral administration olive oil (1 mL) with or without DE supplementation in healthy female Wistar rats. Data are expressed as mean ± SEM. C: rats administered daily with tap water; DE2: rats supplemented with 2 mg silicon/kg body weight/day; DE4: rats supplemented with 4 mg silicon/kg body weight/day. (**A**) Total gastric content (g). (**B**) Gastric fat content (g/100g), (**C**) Triglyceride concentration in gastric content (g/100g fat). (**D**) Diglyceride concentration in gastric content (g/100g fat). (**E**) Free fatty acids concentration in gastric content (g/100g fat). Fat composition was analyzed using HPSEC. * indicates statistically significant differences between groups (* *p* < 0.05, *** *p* < 0.001) determined by one-way ANOVA followed by Bonferroni or Tamhane post hoc test, as appropriate.

**Figure 3:**
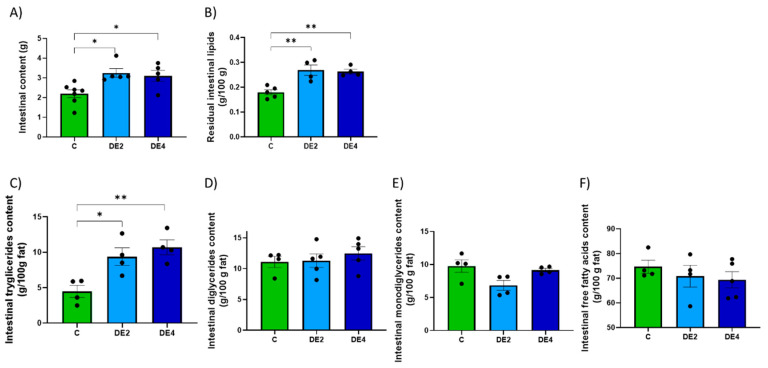
Intestinal content and lipid composition after 2.5 h of an oral administration olive oil (1 mL) with or without DE supplementation in healthy female Wistar rats. Data are expressed as mean ± SEM. C: rats administered daily with tap water; DE2: rats supplemented with 2 mg silicon/kg body weight/day; DE4: rats supplemented with 4 mg silicon/kg body weight/day. (**A**) Total intestinal content (g), (**B**) Intestinal fat content (g/100g), (**C**) Triglyceride concentration in intestinal content (g/100g fat). (**D**) Diglyceride concentration in intestinal content (g/100g fat), (**E**) Monoglyceride concentration in intestinal content (g/100g fat). (**F**) Free fatty acid concentration in intestinal content (g/100g fat). Fat composition was analyzed using HPSEC. * indicates statistically significant differences between groups (* *p* < 0.05, ** *p* < 0.01) determined by one-way ANOVA followed by Bonferroni or Tamhane post hoc test, as appropriate.

Including DE in olive oil as part of a lipid overload significantly affected both gastric and intestinal remanent contents, as well as their lipid composition (Figure 2 and Figure 3). In the stomach (Figure 2A–E), the content weight was significantly reduced in both DE2 (−33.4%, *p* < 0.001) and DE4 (−33.4%, *p* < 0.001) groups compared to C group (Figure 2A), while residual gastric lipids increased significantly in DE2 (+80.3%, *p* < 0.001) and even further in DE4 (+128.0%, *p* < 0.001) (Figure 2B). Despite this, no significant differences were observed in the relative composition of TG, DG, or FFA in gastric contents (Figure 2C–E). In the intestine (Figure 3A–F), intestinal content weight (DE2 = +47.7%, *p* = 0.017; DE4 = +40.9%, *p* = 0.042) (Figure 3A) and residual intestinal lipids (DE2 = +49.6%, *p* = 0.0032; DE4 = +46.6%, *p* = 0.0049) (Figure 3B) were significantly increased in the DE groups compared to C. Intestinal TG content was markedly elevated in both DE groups compared to C group (DE2 = +109.5%, *p* = 0.0298; DE4 = +139.1%, *p* = 0.0076) (Figure 3C), with no further increase between DE2 and DE4. However, no significant differences were found among groups in intestinal DG, MG, or FFA (Figure 3D–F).

### 3.4. Morphometric Parameters and Mucin Production in the Jejunal Mucosa

**Figure 4:**
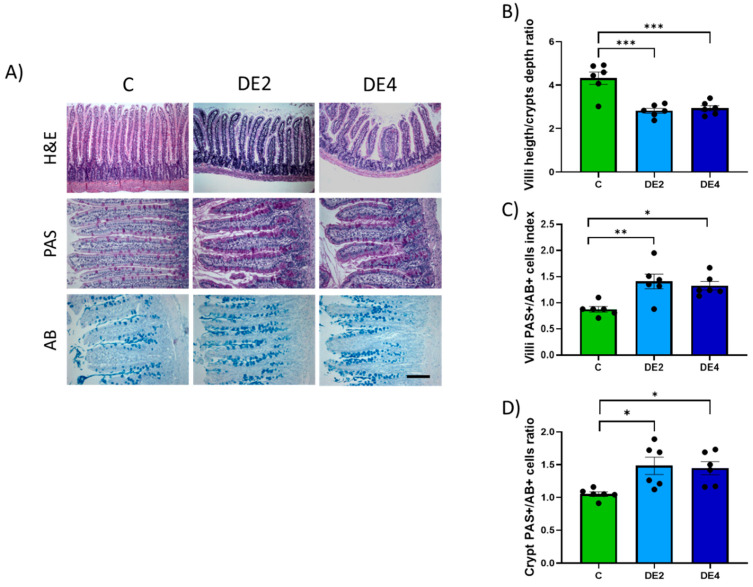
Histological analysis of jejunal architecture and mucin-producing cells in DE-supplemented healthy female Wistar rats. Data are expressed as mean ± SEM. C: rats administered daily with tap water; DE2: rats supplemented with 2 mg silicon/kg body weight/day; DE4: rats supplemented with 4 mg silicon/kg body weight/day. (**A**) Representative micrographs of jejunal sections stained with hematoxylin and eosin (H&E) (scale bar: 250 µm), used for villus and crypt morphology. Periodic acid–Schiff (PAS), for detecting neutral mucins and alcian blue (AB) (scale bar: 50 µm), for detecting acidic mucins. (**B**) Villus-to-crypt ratio based on H&E-stained sections (**C**) Villi PAS/AB+ cells index and (**D**) PAS/AB+ cells index in crypts only. * indicates statistically significant differences between groups (* *p* < 0.05, ** *p* < 0.01, *** *p* < 0.001) determined by one-way ANOVA followed by Bonferroni or Tamhane post hoc test, as appropriate.

Figure 4A–D displays the villus height to crypt depth ratio and the PAS/AB+ cells index in the jejunal epithelium. Photographs representing the H&E, PAS, and AB staining (Figure 4A). The C group exhibited the highest villus height-to-crypt-depth ratio, while the DE2 and DE4 treated groups showed reductions of −34.8% (*p* = 0.0002) and −32.1% (*p* = 0.0004), respectively, compared to the C group (Figure 4B), with no significant changes in crypt depth. In contrast, the PAS/AB+ cells index significantly increased in both the villi (Figure 4C) and crypts (Figure 4D) of the DE-treated groups with respect to the C group (Villi: DE2 = +60.6%, *p* = 0.005; DE4 = +51.1%, *p* = 0.017. Crypts: DE2 = +41.1%, *p* = 0.020; DE4 = +37.6%, *p* = 0.035), indicating enhanced mucin production, which may reflect a compensatory or protective response of the intestinal epithelium to the treatment, without being dose-dependent.

### 3.5. Jejunal Lipid Absorption Markers

**Figure 5:**
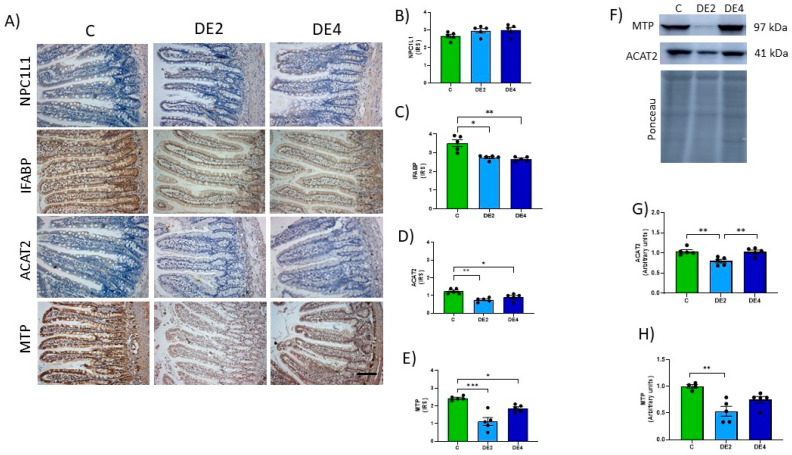
Jejunal levels of the proteins involved in lipid absorption: NPC1L1, Niemann–Pick C1-Like 1; IFABP, intestinal fatty acid-binding protein; ACAT2, acetyl-Coenzyme A acetyltransferase-2; MTP, microsomal triglyceride transfer protein of the jejunal mucosa in healthy female Wistar rats. Values expressed as mean ± SEM. C: rats administered daily with tap water; DE2: rats supplemented with 2 mg silicon/kg body weight/day; DE4: rats supplemented with 4 mg silicon/kg body weight/day. (**A**) Representative images of immunohistochemistry labeling of NPC1L1, IFABP, ACAT2, and MTP. Scale bar: 50 µm. Immunoreactivity scores (IRS) of (**B**) NPC1L1, (**C**) IFABP, (**D**) ACAT2 and (**E**) MTP. (**F**) Bands of Western blot and data of densitometric quantification for jejunal, (**G**) ACAT2, and (**H**) MTP. * indicates statistically significant differences between groups (* *p* < 0.05, ** *p* < 0.01, *** *p* < 0.001) determined by the one-way Kruskal–Wallis test, followed by the Benjamini–Krieger–Yekutieli post hoc or ANOVA followed by Bonferroni or Tamhane post hoc test, as appropriate.

Markers of jejunal lipid absorption are shown in Figure 5A–H. Photographs representing the immunostaining (Figure 5A) and IRS of Niemann–Pick C1-like 1 (NPC1L1) (Figure 5B), intestinal fatty acid-binding protein (IFABP) (Figure 5C), acetyl-CoA acetyltransferase 2 (ACAT2) (Figure 5D), and microsomal TG transfer protein (MTP) (Figure 5E) levels. ACAT2 and MTP levels also were assayed by Western blot (Figure 5F–H).

No significant differences were observed in NPC1L1 levels between C and DE groups (Figure 5B). In contrast, the IFABP level (Figure 5C) was significantly reduced in DE-treated animals (DE2 = −21.8%, *p* = 0.0284; DE4 = −24.1%, *p* = 0.0019) compared to C rats. Likewise, ACAT2 immunochemistry analysis showed a significant reduction in IRS in both DE-treated groups compared to the control one: −38.7% in DE2 (*p* = 0.0017) and −25.8% in DE4 (*p* = 0.0459). Western blot analysis confirmed a significant decrease only in the DE2 group (−23.2%, *p* = 0.0058), while no change was observed in DE4 (*p* > 0.05). For MTP, IRS values also showed a marked reduction: −54.1% in DE2 (*p* = 0.0008) and −23.8% in DE4 (*p* = 0.0514), the latter showing a trend toward significance. Similarly, WB data revealed a significant decrease in DE2 (−46.7%, *p* = 0.0025), whereas the reduction in DE4 (−25.0%) did not reach statistical significance (*p* = 0.090).

### 3.6. Jejunal Cholesterol Efflux Markers

**Figure 6:**
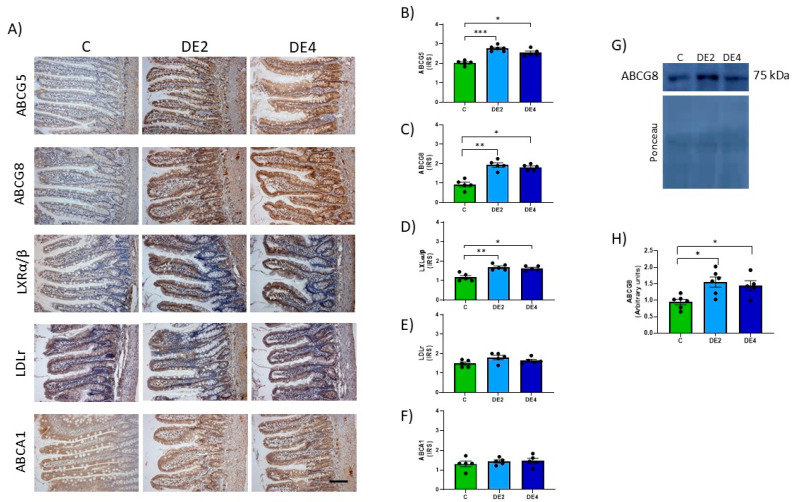
Jejunal levels of the proteins involved in cholesterol efflux: ATP-binding cassette subfamily G members 5 (ABCG5) and 8 (ABCG8) and subfamily A1 (ABCA1) transporters, the liver X receptor transcription factor (LXRα/β) and low-density lipoprotein receptor (LDLr) of the jejunal epithelium in healthy female Wistar rats. Values expressed as mean ± SEM. C: rats administered daily with tap water; DE2: rats supplemented with 2 mg silicon/kg body weight/day; DE4: rats supplemented with 4 mg silicon/kg body weight/day. (**A**) Representative images of immunohistochemistry labeling. Scale bar: 50 µm. Immunoreactivity scores (IRS) of (**B**) ABCG5, (**C**) ABCG8, (**D**) LXRα/β, (**E**) LDLr, and (**F**) ABCA1. (**G**) Band of Western blot and (**H**) percentage data of densitometric quantification for jejunal ABCG8. * indicates statistically significant differences between groups (* *p* < 0.05, ** *p* < 0.01, *** *p* < 0.001) determined by the one-way Kruskal–Wallis test, followed by the Benjamini–Krieger–Yekutieli post hoc or ANOVA followed by Bonferroni or Tamhane post hoc test, as appropriate.

Figure 6A–H shows the photographs and IRS of jejunal ATP-binding cassette subfamily G members 5 (ABCG5) and 8 (ABCG8), liver X receptor transcription factor (LXRα/β), low-density lipoprotein receptor (LDLr) and ATP-binding cassette subfamily A1 (ABCA1) immunohistochemical detection. Figure 6G–H represent the ABCG8 blots and levels measured by Western blot. DE groups presented significantly higher IRS of ABCG5 (DE2 = +36.5%, *p* = 0.0008; DE4 = +25.8%, *p* = 0.0420), ABCG8 (IRS: DE2 = +107.5%, *p* = 0.0028; DE4 = +94.6%, *p* = 0.019; WB: DE2 = +63.5%, *p* = 0.0113; DE4 = +52.2%, *p* = 0.046) and LXRα/β (DE2: +44.4%, *p* = 0.0036; DE4: +39.3%, *p* = 0.0156) with respect to the C group. Non-significant differences in IRS of LDLr (Figure 6E) and ABCA1 (Figure 6F) proteins in DE groups compared to their C counterpart were observed.

### 3.7. Hepatic Cholesterol Metabolism Markers

**Figure 7:**
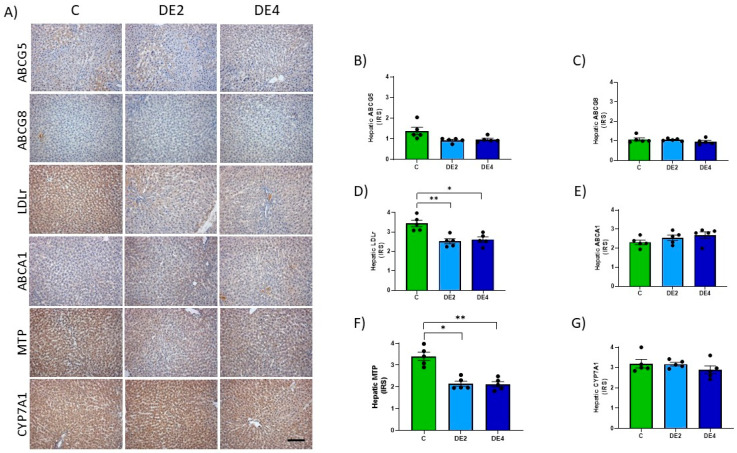
Hepatic levels of proteins involved in lipid uptake and secretion: ATP-binding cassette subfamily G members 5 (ABCG5) and 8 (ABCG8), low-density lipoprotein receptor (LDLr), ATP-binding cassette transporter A1 (ABCA1), microsomal triglyceride transfer protein (MTP) and cholesterol 7 alpha-hydroxylase (CYP7A1) in DE-supplemented healthy female Wistar rats. Values expressed as mean ± SEM. C: rats administered daily with tap water; DE2: rats supplemented with 2 mg silicon/kg body weight/day; DE4: rats supplemented with 4 mg silicon/kg body weight/day. (**A**) Representative immunohistochemical images of liver sections showing immunohistochemistry labeling. Scale bar: 50 µm. Immunoreactivity scores (IRS) of (**B**) ABCG5 and (**C**) ABCG8, (**D**) LDLr, (**E**) ABCA1, (**F**) MTP, and (**G**) CYP7A1. * indicates statistically significant differences between groups (* *p* < 0.05, ** *p* < 0.01,) determined by the one-way Kruskal–Wallis test, followed by the Benjamini–Krieger–Yekutieli post hoc.

Figure 7 shows photographs representing the immunostaining (Figure 7A) and IRS of hepatic ABCG5, ABCG8, LDLr, ABCA1, MTP and cholesterol 7 alpha-hydroxylase (CYP7A1) proteins (Figure 7B–G). No significant differences were found among groups in ABCG5, ABCG8, ABCA1, and CYP7A1 levels. IRS of LDLr (DE2 = −26.6% *p* = 0.0058; DE4 = −24.2%, *p* = 0.0109) (Figure 7D) and MTP (DE2 = −36.9% *p* = 0.0133; DE4 = −37.8%, *p* = 0.0047). (Figure 7F) significantly decreased in both DE groups with respect to the C group.

### 3.8. Heatmap

**Figure 8:**
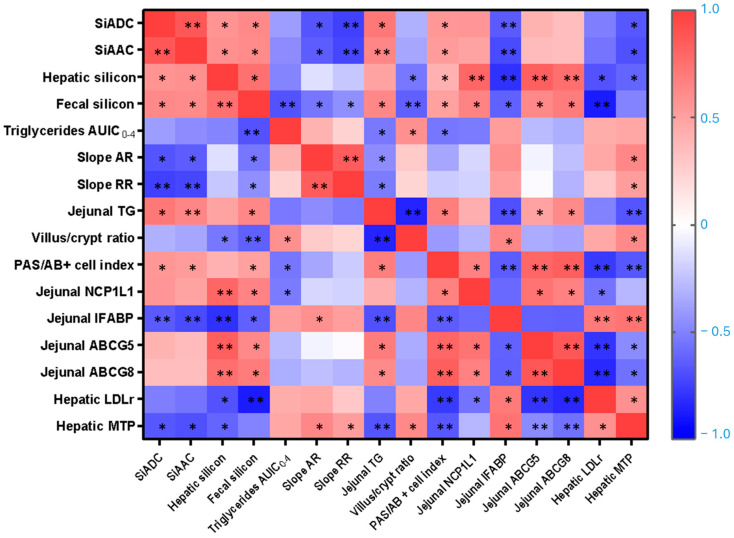
Correlations between silicon-related parameters, lipemic status, jejunal morphometry, mucin production, and jejunal and hepatic cholesterol transporters and enzymes. Pearson’s and Spearman’s correlation values were used for the matrix. The parameters displayed in the heatmap are the silicon apparent digestibility coefficient (SiADC), silicon absolute absorption coefficient (SiAAC), hepatic silicon, fecal silicon, triglycerides area under the incremental curve (AUIC_0–4_), absorption rate (AR), removal rate (RR), jejunal triglycerides (TG), villus/crypt ratio, periodic acid-Schiff to alcian blue positive (PAS/AB+) cells index, jejunal Niemann–Pick C1-like 1 (NCP1L1), jejunal intestinal fatty acid-binding protein (IFABP), jejunal ATP-binding cassette subfamily G members 5 (ABCG5) and 8 (ABCG8), hepatic low-density lipoprotein receptor (LDLr), hepatic microsomal triglyceride transfer protein (MTP). The color intensity of the heatmap represents the association degree: red for positive, and blue for negative. * *p* < 0.05, ** *p* < 0.01.

Figure 8 summarizes all significant Pearson’s or Spearman’s correlations among the main markers of silicon bioavailability, lipid metabolism, and intestinal and hepatic parameters assessed in this study. Significant differences are highlighted and marked with asterisks (* *p* < 0.05, ** *p* < 0.01).

To explore whether silicon bioavailability predicts its hypolipidemic effect, silicon bioavailability indicators, measured as SiADC and SiAAC, were correlated with AUIC_0–4_ and AR and RR. Both SiADC and SiAAC were significantly and negatively correlated with the AR (r = −0.636 and −0.664; *p* = 0.035 and 0.026, respectively) and the RR (r = −0.718 and −0.745; *p* = 0.013 and 0.008, respectively), indicating an inverse association between silicon availability and lipid uptake. No significant correlation was found between hepatic silicon content and either the AR or RR. In addition, both SiADC and SiAAC positively correlated with hepatic silicon content (r = 0.588 and 0.564; *p* = 0.039and 0.045, respectively), fecal silicon content (r = 0.622 and 0.608; *p* = 0.036 and 0.031, respectively), jejunal TG content (r = 0.709 and 0.636; *p* = 0.022 and 0.048, respectively), and the PAS/AB+ cells index (r = 0.545 and 0.524; *p* = 0.043 and 0.048, respectively), confirming the effective systemic distribution and a mild hepatic accumulation of dietary silicon. Conversely, SiADC and SiAAC showed negative correlations with the levels of jejunal IFABP (r = −0.650 and −0.706; *p* = 0.022 and 0.010, respectively) and hepatic MTP (r = −0.657 and −0.685; *p* = 0.020 and 0.014, respectively). In addition, TG AUIC_0–4_ appears positively associated with villus/crypt ratio (r = 0.582; *p* = 0.029) but negatively with fecal silicon content (r = −0.675; *p* = 0.008), jejunal TG (r = −0.536: *p* = 0.041), and PAS/AB+ cells index (r = −0.543, *p* = 0.045).

The relationship between structural intestinal mucosae, lipid trafficking within the intestine, and liver was supported by a strong negative correlation between the villus/crypt ratio and jejunal TG levels (r = −0.846; *p* < 0.001), and a positive correlation with jejunal IFABP levels (r = 0.643; *p* = 0.01). Notably, intestinal IFABP levels were also significantly and positively correlated with hepatic LDLr (r = 0.700; *p* = 0.004) and MTP levels (r = 0.729; *p* = 0.002). Furthermore, hepatic silicon levels correlated positively with NPC1L1 (r = 0.798, *p* = 0.001) and jejunal ABCG5/G8 (r = 0.836 and 0.759, *p* = 0.0001 and 0.003, respectively) and negatively with villus/crypt ratio (r = −0.541; *p* = 0.03), jejunal IFABP (r = −0.797, *p* = 0.001) and hepatic LDLr and MTP (r = −0.676 and −0.610; *p* = 0.011 and 0.027, respectively). Both fecal silicon content and jejunal TG were inversely correlated with the villus/crypt ratio and intestinal IFABP levels, while positively correlated with the PAS/AB+ cells index and ABCG5/G8 levels (*p* < 0.01 to *p* < 0.001). Additionally, negative correlations were observed between fecal silicon and the hepatic LDLr level (r = −0.871; *p* < 0.001) and between jejunal ABCG5/G8 and hepatic MTP levels (r = −0.785 and −0.557; *p* < 0.001 and 0.031, respectively), suggesting compensatory regulation between cholesterol efflux transporters and hepatic lipid export machinery.

## 4. Discussion

In this study, we demonstrated for the first time that short-term oral supplementation with food-grade DE promoted coordinated adaptations in lipid absorption and metabolism in healthy female rats. The observed effects include the following: (a) enhanced silicon digestibility and absorption, supporting the suitability of DE as a bioavailable dietary silicon source; (b) a significant dose-dependent reduction in postprandial triglyceridemia without affecting the glycemic response; (c) modulation of gastrointestinal function, altering gastric emptying and inducing dose-dependent changes in fecal output and moisture content; (d) increased remnant levels of TG in the intestinal content, suggesting reduced fat digestion and the presence of unabsorbed lipids; (e) reductions in villus height and an increase in the number of mucin-producing cells in the jejunal mucosa; (f) downregulation of key proteins involved in intestinal (IFABP, ACAT2, MTP) and hepatic (LDLr, MTP) lipid metabolism, indicating reduced capacity for lipid transport and absorption; (g) upregulation of the intestinal LXRα/β–ABCG5/G8 pathway, in the absence of changes in hepatic cholesterol efflux markers. These findings position DE as a promising dietary supplement or functional ingredient with potential applications in lipid modulation and metabolic disorders.

Our findings showed that short-term oral supplementation with DE represents a bioavailable source of silicon in healthy female rats. After only one week of treatment, both SiADC and SiAAC increased in a dose-dependent manner, indicating efficient gastrointestinal digestibility and absorption. The increased fecal silicon in DE-fed animals reflects both higher intake and incomplete absorption, consistent with typical in vivo silicon dioxide digestibility [26]. Simultaneously, significantly higher silicon concentrations in the liver confirmed effective systemic absorption. The positive correlations between SiADC and SiAAC with hepatic silicon levels (r = 0.564 and 0.588; *p* = 0.045 and 0.039, respectively) further support the systemic distribution and tissue deposition of dietary silicon freed from DE. However, the absence of a dose-dependent increase in hepatic silicon content may suggest the existence of a physiological saturation threshold or a homeostatic mechanism that limits hepatic absorption. This is consistent with previous research indicating that the liver is not a primary regulatory organ for silicon but may instead act as a transit site or temporary storage compartment [31]. Importantly, these results support the hypothesis that the silicon absorption observed after DE supplementation is related to its physicochemical nature [8]. Although amorphous silica is water-insoluble, it becomes hydrated in the gastrointestinal environment and is subsequently converted into OSA, the bioavailable form of silicon [6,7]. Moreover, the lack of detectable silicon in plasma supports the hypothesis of rapid tissue uptake or efficient systemic clearance, as proposed in earlier pharmacokinetic models [26]. This is further reinforced by the known properties of silicon, including its rapid removal from the bloodstream and low affinity to plasma binding proteins, which facilitate its efficient renal filtration and excretion [8,9]. Although urinary silicon excretion is commonly used as a marker of absorption and bioavailability, no significant differences were observed between groups in this study. This outcome likely reflects the timing of urine collection, which began immediately after the final administration of water with or without DE. Although urine was collected over a 24 h period, silicon is known to be rapidly excreted, with peak urinary elimination occurring within 6–12 h post-ingestion [26]. As a result, the excreted silicon may have been diluted over the 24 h collection period, leading to similar urinary silicon levels across all groups and potentially underestimating short-term excretion differences.

DE supplementation induced a marked and dose-dependent reduction in postprandial triglyceridemia. These findings are particularly relevant considering that the OTTT is a validated method to assess postprandial lipid metabolism and is widely employed as a marker of cardiometabolic risk [25]. The DE2 group showed significant reductions at 2 and 3 h, whereas the DE4 group exhibited sustained decreases at 2, 2.5, and 3 h, as well as in AUIC_0–4_ and AUIC_2_._5–4_ (*p* < 0.05), suggesting a beneficial effect on lipid metabolism. These beneficial effects occurred without changing AR slopes but with a significant decrease in the RR slope in DE4 rats compared to both the control and DE2 animals. This suggests that DE4 supplementation modifies postprandial lipid dynamics, potentially through a shift in systemic handling or tissue distribution of lipids [31]. Hence, DE4, rather than DE2, exerted a more pronounced postprandial triglycerides-lowering effect, reinforcing the idea of a dose-dependent response. The stronger hypotriglyceridemic effect observed in the DE4 group could be linked to silicon bioavailability, as evidenced by significant negative correlations of SiADC and SiAAC with both AR and RR. These relationships suggest that silicon may contribute to reduce and delay lipid uptake, possibly regulating triglyceride (TG) clearance more effectively [25], thereby underscoring its potential role as a key factor responsible for the lipid-lowering effects of DE. In line with these findings, silicon has been shown to reduce postprandial triglyceridemia from the first administration and delay the maximum peak, suggesting a direct effect on lipid digestion or absorption [13]. Similarly, Najda et al. [31] observed silicon-induced hypotriglyceridemia in healthy rats, attributing this effect to altered activity of lipid-degrading enzymes such as lipoprotein lipase. Previous studies have shown that silicon supplementation, whether in amorphous form [24] or incorporated as monomethylsilanetriol and stabilized OSA into a food matrix [15], can attenuate diet-induced triglyceridemia in animal models of metabolic syndrome. This effect was associated with higher fecal fat excretion, likely reflecting lower lipid digestion and absorption [18,19]. These findings are particularly noteworthy given the physiological characteristics of female rodents, who typically show an attenuated postprandial triglyceride response compared to males [16]. This sex-related metabolic profile is influenced by hormonal factors such as estrogens, which modulate hepatic lipid metabolism by enhancing HDL production, reducing VLDL secretion, and promoting peripheral lipid uptake [32]. Moreover, females generally exhibit slower intestinal lipid absorption and higher lipoprotein lipase activity in adipose tissue [33,34], which may attenuate the apparent effects of lipid-lowering interventions. Therefore, the sustained reduction in postprandial TG levels observed in the DE4 group under these physiological conditions provides strong evidence for the metabolic efficacy of DE. These results underline the importance of evaluating therapeutic interventions in female models, which remain underrepresented in preclinical metabolic studies despite clear sex-based differences in lipid regulation.

To help explain the mechanisms underlying silicon’s lipid-lowering effect, current evidence first points to its interactions within the gastrointestinal tract, where it may interfere with lipid emulsification and absorption processes [16,22,24]. Both DE doses modified overall gastric emptying, promoting earlier delivery of nutrients to the intestine. However, lipid emptying was selectively delayed, leading to fat retention in the stomach. This dissociation likely impaired fat digestion and could explain the elevated intestinal TG levels observed in DE-fed rats, indicating the presence of unabsorbed fat. Importantly, levels of DG, MG, and FFA remained unchanged, suggesting that DE selectively blocked the first step of TG hydrolysis, likely through inhibition of pancreatic lipase [31,35,36]. Notably, the positive correlation between SiADC and SiAAC, fecal silicon concentration, and intestinal TG content suggests that silicon may transiently modulate lipid absorption or retention in the intestine, reinforcing the hypothesis of a functional interaction occurring within the intestinal lumen. This observation aligns with previous reports indicating that DE could interfere with lipid emulsification or absorption, possibly by affecting micelle formation or digestive enzyme activity [23]. This could explain the delayed lipid transport and reduced postprandial triglyceridemia observed in DE-fed groups. Silicon particles may also interfere with bile salts and dietary lipids, altering micelle formation and intestinal function [37]. Similar mechanisms have been described for other dietary compounds or mineral particles that block enzyme access to lipid droplets [38,39]. In vitro studies have shown that mesoporous silica [40], as well as the addition of DE to gelled emulsions [23] or orally administered amorphous silicon particles, reduces lipid digestibility by stabilizing the emulsion matrix and limiting enzyme access, thereby further inhibiting micelle formation [41,42].

In line with these effects, we also found that at the highest dose tested, DE significantly increased fecal moisture and dry matter excretion, likely due to its hygroscopic and osmotic properties [5], which promote water retention in the intestinal lumen and alter stool consistency. Importantly, despite the increase in fecal moisture, stools remained non-diarrheic, suggesting that DE did not induce a laxative effect under the conditions of this study. These effects are consistent with previous findings showing that amorphous silicon can influence gastrointestinal transit and defecation patterns by retaining water and interacting with the intestinal environment [43]. Moreover, amorphous silicon has been shown to accumulate in the intestinal lumen due to its low absorption rate, prolonging its contact time and physiological activity within the gut [44]. The accumulation of unabsorbed lipids alongside silicon may contribute to increased osmotic load and water retention in the intestinal lumen, further explaining the changes in fecal consistency and output. Previous studies have shown that unabsorbed dietary fat can affect water balance, transit time, and intestinal motility [45]. However, since feces were not collected on the day of the lipid overload, the fecal data likely reflect cumulative rather than acute effects.

The hypotriglyceridemic action of DE may be attributed not only to impaired micellar emulsification and limited enzymatic access in the intestinal lumen, but also to structural and biochemical remodeling of the intestinal mucosa that may affect lipid uptake. Morphometric analysis revealed moderate villus shortening without changes in crypt depth, preserving overall mucosal structure. Since villi increase the surface area for micelle–enterocyte interaction, their reduction impairs lipid absorption [46]. This change is reflected by the strong negative correlation between villus crypt ratio and both intestinal TG levels and fecal silicon content (r = −0.846, *p* = 0.0001; r = −0.624, *p* = 0.0006), suggesting reduced lipid uptake due to decreased absorptive surface. This interpretation is consistent with previous reports demonstrating that in vitro exposure to silicon can decrease the number of intestinal microvilli, thereby reducing the effective absorptive surface [47]. In parallel, DE-supplemented rats showed an increase in the number of mucin-producing cells, as evidenced by villus and crypt PAS/AB+ cells indexes. While mucins are essential for maintaining epithelial barrier function and protecting against luminal stressors, excessive secretion can act as a mechanical barrier to nutrient absorption [20]. Specifically, a thickened mucus layer may hinder micelle diffusion across the unstirred water layer, limiting their access to the apical surface of enterocytes and further impairing lipid uptake [48,49]. This is supported by the positive correlation observed between jejunal TG content and the villus PAS/AB+ cells index (r = 0.680, *p* = 0.015), suggesting that increased mucin production may influence luminal lipid dynamics. The combination of reduced surface area and increased mucin production establishes a robust barrier to lipid uptake, which mechanistically supports the observed increase in luminal TG retention and the concomitant decrease in systemic lipid levels. This is further supported by the significant correlations (*p* < 0.05) observed between the villus/crypt ratio and villi PAS/AB+ cells index with intestinal TG content, fecal silicon content and the AUIC_0–4_. Notably, the number of PAS+ cells exceeded that of AB+ cells, indicating a predominance of neutral mucins. This pattern is generally associated with a healthy mucosal state, as neutral mucins contribute to baseline epithelial protection and are upregulated in response to physiological stimuli rather than inflammatory stimuli [49]. Taken together, these findings suggest that both mucin upregulation and moderate villus shortening represent adaptive responses to DE exposure rather than adverse effects. The enhancement of neutral mucin production, along with preserved crypt architecture and no evidence of inflammation or inflammatory signs, supports the interpretation that the intestinal epithelium is undergoing a protective remodeling process to maintain barrier integrity under the influence of non-absorbable silicon from DE. In this regard, a recent study using a murine model of intestinal ischemia–reperfusion injury demonstrated that silicon-based treatment preserved goblet cells and maintained mucin output, contributing to epithelial integrity and gut barrier function [50]. Similarly, increased mucin production and villus shortening have also been observed throughout all intestinal segments in diabetic rats fed a Si-RM, suggesting a generalized mucosal response rather than a regionally restricted effect [19,21]. Although these changes were more pronounced at the higher DE dose, the absence of histological damage or signs of inflammation supports their classification as compensatory rather than harmful. These findings align with evidence from studies on amorphous silica nanoparticles (ASNP), which indicate that although only a small fraction of ASNP is absorbed, most remain in contact with the intestinal wall, where they can exert local effects on epithelial and immune cells [44]. Such changes may influence intestinal homeostasis and nutrient handling, with broader implications for metabolic health.

To further explore the mechanisms underlying the hypolipemic effect of DE, we investigated key proteins involved in lipid metabolism in the jejunum and the liver. Our findings indicate that DE modulated transporters and markers associated with lipid absorption and cholesterol transport, suggesting a broader role in regulating systemic lipid homeostasis beyond the intestinal lumen. These results are even more important, taking into account that the sacrifice of experimental rats was carried out in the postprandial period, when the analyzed markers are physiologically upregulated.

DE supplementation led to the downregulation of key proteins involved in lipid absorption in the jejunum, including IFABP, ACAT2, and MTP. The concurrent reduction in IFABP and ACAT2 levels suggest a decrease in intracellular fatty acid trafficking and a reduced capacity for cholesterol esterification within enterocytes, consistent with reduced lipid absorption efficiency. The downregulation of MTP, a key protein required for chylomicron assembly and lipid export, suggests that DE may reduce intestinal lipid output into circulation, thus contributing to its postprandial hypotriglyceridemic effect [48]. This supports a mechanistic link between DE and decreased enterocyte-mediated lipid transport. Additionally, the downregulation of MTP implies a reduced capacity for chylomicron assembly and secretion, further supporting the intestinal contribution to DE’s postprandial hypotriglyceridemic effect [51]. In contrast, the LXRα/β-ABCG5/G8 pathway was significantly upregulated, indicating activation of cholesterol efflux pathways. This pathway facilitates direct excretion of free cholesterol from enterocytes into the intestinal lumen, contributing to intestinal cholesterol homeostasis [52]. The upregulation of ABCG5/G8 transporters is particularly relevant, as these proteins are involved in limiting dietary cholesterol absorption and promoting sterol elimination, potentially preventing intestinal lipid accumulation. Significant correlations between IFABP and ABCG5/G8 expression levels with fecal and hepatic silicon contents, as well as with intestinal TG content, have been found (*p* < 0.05). These findings support the hypothesis that DE supplementation modulates intestinal lipid homeostasis by downregulating key proteins involved in lipid absorption while simultaneously activating cholesterol efflux mechanisms. However, no significant changes were observed in jejunal LDLr levels, suggesting that DE did not modulate transintestinal cholesterol excretion (TICE) at least in healthy animals [53]. This may reflect sufficient intracellular cholesterol levels resulting from increased efflux via ABCG5/G8, eliminating the need for additional LDLr-mediated uptake/activation of transintestinal cholesterol excretion (TICE) [54]. On the other hand, no significant changes were observed in ABCA1 levels in either the intestine or liver, suggesting that DE did not influence reverse cholesterol transport (RCT) [55]. This is consistent with the physiological context of the study, which involved healthy rats with an intact lipid metabolism. In the liver, DE supplementation reduced hepatic MTP and LDLr levels, which would lead to a restriction in VLDL production, thereby eliminating the need for increased hepatic uptake [56]. This coordinated hepatic downregulation of MTP and LDLr may reflect a shift toward reduced lipid export and peripheral uptake, contributing to a more cardioprotective lipoprotein profile [54,55]. The unchanged levels of ABCA1, ABCG5/G8, and CYP7A1 confirm that cholesterol efflux via HDL formation, biliary secretion, and bile acid synthesis remained stable [57]. This reduction in VLDL in healthy female rats mirrors previous findings in a male model of advanced T2DM, where Si-RM similarly reduced MTP while upregulating ABCA1, LDLr, and ABCG5/G8, thereby improving the TICE and RCT pathways [18]. Taken together, the data suggest that DE modulates lipid metabolism through context-dependent intestinal and hepatic mechanisms that warrant further investigation.

Considering all the results of this study, although silicon bioavailability increased dose-dependently, this was not consistently reflected in all measured outcomes. The 4 mg Si/kg body weight/day dose had the strongest effect on postprandial TG, but other variables did not show a clear dose–response. This suggests DE acts mainly through luminal intestinal mechanisms, which may reach a saturation threshold. Thus, DE4 appears optimal doses in this model. Future studies should determine the minimum effective dose and further explore local and systemic mechanisms.

On the other hand, DE supplementation did not produce significant changes in postprandial glucose levels in this study. However, previous research has reported hypoglycemic effects with other forms of dietary silicon. Silicon supplementation reduced glucose and TG digestion and absorption, with stronger effects after one week of treatment [13]. Similar results have been reported in diabetic or insulin-resistant models using silicon-enriched matrices, where the hypoglycemic effects were related to improved insulin sensitivity and modulation of carbohydrate metabolism [18,24,31]. Although the absence of a hypoglycemic effect in this study could be linked to the specific formulation and physicochemical properties of DE, it can also be due to the experimental design. DE appears to act mainly in the intestinal lumen, interfering with lipid digestion. Thus, it would be hypothesized that silicon may also interfere with complex carbohydrate digestion in a way that modifies postprandial glycemia. Furthermore, this study was conducted in female rats, unlike most prior studies which used males, suggesting possible sex-specific responses. These findings highlight the importance of silicon formulation, sex, and physiological context in shaping metabolic effects and underscore the need for comparative studies across silicon sources.

This study has some limitations that should be considered in the context of its translational application. (1) The findings are based on a short-term intervention in healthy rats fed a standard chow diet. (2) The use of only female rats may limit generalizability across sexes. (3) The comparative efficacy of DE versus other silicon sources should be addressed in future studies. (4) Although urine was collected over 24 h following standard protocols, shorter collection intervals could provide a more accurate estimate of silicon bioavailability. Despite these limitations, some strengths can be highlighted. This is the first time the effects of DE-derived silicon have been explored in healthy female rat models. The inclusion of two different doses allowed for the identification of dose-dependent effects. Analyzing markers involved in digestion and absorption during postprandial state provides novel insights, as previous studies have primarily focused on fasting conditions. Taken together, the observed effects on lipid metabolism and intestinal function underscore the potential of DE as a novel nutritional strategy for managing postprandial hyperlipidemia.

## 5. Conclusions

Short-term supplementation with food-grade DE ensured silicon absorption and induced coordinated gastrointestinal and metabolic adaptations in healthy female rats, leading to a reduction in postprandial triglyceridemia. DE remodeled the intestinal mucosa, downregulated proteins involved in lipid absorption, and promoted cholesterol efflux. Hepatic responses were consistent with these intestinal changes, showing reduced lipid uptake and cholesterol secretion without activation of compensatory clearance pathways. These findings highlight the potential of DE as a supplement or functional ingredient for the dietary management of lipid metabolism. Further studies are needed to confirm these effects, clarify the underlying mechanisms, and evaluate its efficacy in pathological models associated with dyslipidemia or metabolic disorders.

## Figures and Tables

**Table 1:** Ponderal parameters, fecal excretion, fecal moisture, and urine excretion; and hepatic, fecal, and urine silicon contents; and silicon apparent digestibility and absolute absorption coefficients of control (C), 2 mg silicon /kg body weight (DE2), and 4 mg silicon /kg body weight (DE4) groups.

	C	DE2	DE4	*p*
Body weight increase (g)	−0.71 ± 4.41	2.50 ± 5.58	3.83 ± 8.03	NS
Stomach weight (g)	1.32 ± 0.11	1.42 ± 0.11	1.41 ± 0.16	NS
Small intestine weight (g)	5.90 ± 0.96	6.68± 0.70	6.43 ± 1.00	NS
Small intestine length (cm)	101.8 ± 3.90	107.3 ± 6.40	103.1 ± 7.00	NS
Liver weight (g)	5.35 ± 0.44	6.13 ± 0.48	5.59 ± 0.74	NS
Fecal excretion (24 h) (g dry matter)	1.56 ± 0.24	1.74 ± 0.29	2.02 ± 0.36 *	0.045
Fecal moisture (24 h) (%)	46.10 ± 1.75	46.70 ± 4.10	50.84 ± 1.75 *#	0.016
Urine volume (24 h) (mL)	18.70 ± 9.20	15.50 ± 5.10	17.50 ± 3.80	NS
Hepatic silicon content (µg/g)	1.89 ± 0.71	10.73 ± 3.40 *	8.18 ± 1.67 *	<0.001
Fecal silicon content (µg/g dry matter)	1.67 ± 0.26	3.37 ± 0.79 *	3.55 ± 0.87 *	<0.001
Urine silicon (µg/g)	0.22 ± 0.08	0.22± 0.04	0.22 ± 0.04	NS
SiADC (%)	5.43 ± 2.11	29.46 ± 6.48 *	44.14 ± 9.98 *#	<0.001
SiAAC (mg)	0.36 ± 0.43	2.23 ± 0.92 *	4.25 ± 0.91 *#	0.0002

C: rats administered daily with tap water; DE2: rats supplemented with 2 mg silicon/kg body weight/day; DE4: rats supplemented with 4 mg silicon/kg body weight/day. Values are presented as mean ± SD. NS indicates no significant differences between groups. * denotes significant differences respect to the control (C) group; # denotes significant differences respect to the 2 mg silicon/kg body weight (DE2) group (at least *p* < 0.05, determined by ANOVA followed by Bonferroni or Tamhane post hoc test). SiADC, silicon apparent digestibility coefficient, SiAAC, Silicon absolute absorption coefficient.

## Data Availability

The dataset supporting this study is publicly available in the Zenodo repository under the Creative Commons Zero (CC0 1.0) license: https://doi.org/10.5281/zenodo.15786794.

## Abbreviations

The following abbreviations are used in this manuscript:

AB	Alcian blue
ABCA1	ATP-binding cassette subfamily A1
ABCG5	ATP-binding cassette subfamily G member 5
ABCG8	ATP-binding cassette subfamily G member 8
ACAT2	Acetyl-CoA acetyltranferase 2
ANOVA	Analysis of the variance
AR	Absorption rate
ASNP	Amorphous silica nanoparticles
AUIC	Area under the incremental curve
CYP7A1	Cholesterol 7 alpha-hydroxylase
DAB	3,3′-diminobenzidine
DE	Diatomaceous earth
DG	Diglycerides
FAAS	Atomic absorption spectroscopy
FFA	Free fatty acids
H&E	Hematoxylin and eosin
HPSEC	High-performance size-exclusion chromatography
ICP-OES	Inductively coupled plasma optical emission spectrometry
IFABP	Fatty acid-binding protein
IRS	Immunoreactivity score
LDLr	Low-density lipoprotein receptor
LxR	Liver X receptor transcription factor
MG	Monoglycerides
MTP	Microsomal triglyceride transfer protein
NPC1L1	Niemann-Pick C1-like 1
OGTT	Oral glucose tolerance test
OTTT	Oral triglycerides tolerance test
PAS	Periodic acid-Schiff
RCT	Reverse cholesterol transport
RR	Removal rate
SiAAC	Silicon absolute absorption coefficient
SiADC	Silicon apparent digestibility coefficient
SI-RM	Silicon-enriched restructured meat
T2DM	Type 2 Diabetes Mellitus
TG	Triglycerides
THF	Tetrahidrofurane
TICE	Transintestinal cholesterol excretion
